# Piceatannol markedly upregulates heme oxygenase-1 expression and alleviates oxidative stress in skeletal muscle cells

**DOI:** 10.1016/j.bbrep.2019.100643

**Published:** 2019-05-01

**Authors:** Shiori Nonaka, Shinpei Kawakami, Hiroko Maruki-Uchida, Sadao Mori, Minoru Morita

**Affiliations:** Research and Development Institute, Health Science Research Center, Morinaga & Co., Ltd., 2-1-1 Shimosueyoshi, Tsurumi-ku, Yokohama, Kanagawa, 230-8504, Japan

**Keywords:** Piceatannol, Skeletal muscle cell, HO-1, Oxidative stress, Antioxidant enzymes

## Abstract

Piceatannol (PIC), a phytochemical, is abundant in passion fruit (*Passiflora edulis*) seeds. In this study, we investigated the effects of PIC on the expression levels of antioxidant enzymes in C2C12 skeletal muscle cells and compared its effects with those of PIC analogues and polyphenols. We also evaluated its effects on hydrogen peroxide–induced accumulation of reactive oxygen species in C2C12 myotubes. Treatment with PIC led to dose-dependent upregulation of heme oxygenase-1 (*Ho-1*) and superoxide dismutase 1 (*Sod1*) mRNA expression in C2C12 myotubes. PIC was the most potent inducer of *Ho-1* among the PIC analogues and major polyphenols tested. In addition, treatment with PIC suppressed the hydrogen peroxide–induced increase in intracellular reactive oxygen species levels. Our results suggest that PIC protects skeletal muscles from oxidative stress by activating antioxidant enzymes such as HO-1 and SOD1 and can therefore help prevent oxidative stress–induced muscle dysfunction such as muscle fatigue and sarcopenia.

## Highlights

• PIC induced antioxidant enzymes in C2C12 skeletal muscle cell line.
• PIC was the most potent inducer of *Ho-1* among other polyphenols tested.
• Induction potency of PIC for *Sod1* was similar level with those of other polyphenols.
• PIC suppressed the hydrogen peroxide-induced increase in intracellular ROS levels.

## Graphical abstract

**Figure:**
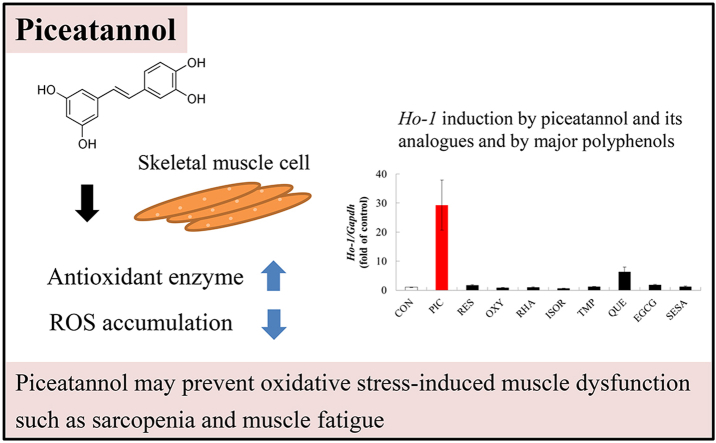

## 1 Introduction

Reactive oxygen species (ROS) are generated by physical exercise and muscle contraction. Physiological levels of ROS, which are generated because of moderate exercise, are important for muscle function [1,2]; however, continuous high-intensity exercises generate high levels of ROS, which promotes skeletal muscle contractile dysfunction resulting in muscle fatigue [3]. In addition, excessive ROS accumulation during aging is suggested to trigger sarcopenia [4].

The Kelch-like ECH-associated protein 1 (Keap1)–nuclear factor erythroid 2–related factor 2 (Nrf2) pathway regulates the gene expression of antioxidant enzymes such as heme oxygenase-1 (*Ho-1*) and superoxide dismutase 1 (*Sod1*); this signaling pathway preserves intracellular redox homeostasis [5]. ROS stimulates redox-sensitive signaling pathways, and activation of antioxidant enzymes prevents oxidative damage to tissues. Imbalances in normal redox states cause oxidative damage, and attenuation of antioxidant activity during aging is reported to contribute to the age-related loss of muscle [6]. Therefore, increasing the antioxidant capacity of skeletal muscles is one of the most valuable therapeutic approaches against for muscle dysfunction [7,8].

Piceatannol (PIC), a natural polyphenolic compound, is present in large amounts in passion fruit (*Passiflora edulis*) seeds [9]. It also presents in red grape and other plants [10]. PIC is an analogue of resveratrol (RES), displays a wide spectrum of biological activities like RES. Our previous studies showed that PIC causes improvement of vascular function [11,12], protects the skin from UV irradiation [13], and promotes *Sirtuin 1* expression [14]. Moreover, PIC has many beneficial effects such as anti-cancer [15] and anti-inflammatory effects [16], and also helps prevent type 2 diabetes [[17], [18], [19]].

PIC has been recently found to suppress aging via activation of Nrf2 and its downstream antioxidant enzyme in the cranial nerve [20]. PIC also activates antioxidant enzymes in tissues other than brain nerves [[21], [22], [23]]. As well as PIC, plant-derived polyphenols are known to upregulate antioxidant enzymes. RES and quercetin (QUE) upregulate *Ho-*1 mRNA expression in astrocytes and microglia [24], and epigallocatechin gallate (EGCG) upregulates *Ho-1* in human aortic endothelial cells [25]. However, the effects of PIC on skeletal muscles have not yet been elucidated. In the present study, we investigated whether PIC induces antioxidant enzymes in a cultured skeletal muscle cell line and compared the effects of PIC with those of PIC analogues and major polyphenols on the induction of antioxidant enzymes. In addition, the effects of treatment with PIC on hydrogen peroxide (H_2_O_2_)-induced ROS accumulation in C2C12 myotubes were evaluated.

## 2 Materials and methods

### 2.1 Materials

PIC, RES, oxyresveratrol (OXY), rhapontigenin (RHA), isorhapontigenin (ISOR), 3,3′,4,5′-tetramethoxypiceatannol (TMP), and sesamin (SESA) were obtained from Tokyo Chemical Industry Co., Ltd. (Tokyo, Japan). Dimethyl sulfoxide (DMSO), EGCG, H_2_O_2_, and *N*-acetylcysteine (NAC) were obtained from Wako Pure Chemical Industries, Ltd. (Osaka, Japan). QUE was obtained from Tocric Co., Ltd. (Bristol, UK). Dulbecco's modified Eagle's medium (DMEM), heat-inactivated horse serum (HS), Hanks' Balanced Salt solution (HBSS), and penicillin and streptomycin solutions were obtained from Gibco (MD, USA). Fetal bovine serum (FBS) was obtained from HyClone (UT, USA). The 5-(and-6) chloromethyl-2′,7′ dichlorodihydrofluorescein diacetate, acetyl ester (CM-H_2_DCFDA) was obtained from Invitrogen (CA, USA).

### 2.2 Cell culture and treatment

C2C12 skeletal muscle cells were obtained from the European Collection of Authenticated Cell Cultures (Salisbury, UK). The cells were grown in DMEM medium containing 10% FBS and 1% penicillin and streptomycin solution in a humidified incubator at 37 °C with 5% CO_2_. When C2C12 myoblast cultures reached confluence, the cells were cultured in DMEM containing 2% HS (differentiation medium), and the mediums were changed every three days. After 6 days, the cells differentiated into myotubes. PIC or the other test compounds were dissolved in DMSO, diluted in differentiation medium to the desired concentration before use, and added to the cell culture. The DMSO concentration in the medium was 0.1% for all conditions. The cytotoxicity was not observed in this treatment.

### 2.3 Real-time PCR analysis of *Ho-1* and *Sod1* mRNA expression

C2C12 myotubes were cultured in 12-well plates and incubated for 6 h in differentiation medium containing different concentrations of PIC or other test compounds. Total RNA was extracted from cells by using the QIAshredder and the RNeasy Mini Kit (QIAGEN, Hilden, Germany) according to the manufacturer's instructions. RNA (1 μg) was reverse transcribed into cDNA by using the High-Capacity cDNA Reverse Transcription Kit (Applied Biosystems, CA, USA). Real-time PCR were performed using a LightCycler 480 Real-Time PCR system II (Roche Molecular Diagnostics, Basel, Switzerland) with the LightCycler 480 Probes Master and Universal Probe Library Probes (Roche Molecular Diagnostics). The assay was performed with probe No. 17 for *Ho-1*, probe No. 49 for *Sod1,* and probe No. 29 for glyceraldehyde 3-phosphate dehydrogenase (*Gapdh*). The PCR primers used were as follows: for *Ho-1*: forward, 5′-aggctaagaccgccttcct-3′ and reverse, 5′-tgtgttcctctgtcagcatca-3′; for *Sod1*: forward, 5′-caggacctcattttaatcctcac-3′ and reverse, 5′-tgcccaggtctccaacat-3′; and for *Gapdh*: forward, 5′-gccaaaagggtcatcatctc-3′ and reverse, 5′-cacacccatcacaaacatgg-3′. The amplification conditions were as follows: 50 °C for 2 min; 95 °C for 10 min; and 45 cycles each of 95 °C for 10 s and 60 °C for 25 s. *Ho-1* and *Sod1* mRNA expression were normalized to *Gapdh* mRNA expression levels, and relative *Ho-1* and *Sod1* mRNA expression were determined in comparison with the corresponding levels in control cells.

### 2.4 Measurement of intracellular ROS levels

C2C12 myotubes were cultured in 96-well plates. After the myotubes were treated with the test compounds for 24 h, they were loaded with CM-H_2_DCFDA (3 μM) at 37 °C under dark condition for 30 min. Subsequently, the cells were washed with HBSS containing 0.2% HS and treated with H_2_O_2_ at 37 °C for 30 min. The fluorescence (excitation/emission at 495 nm/525 nm), reflecting the ROS concentration, was analyzed using a fluorescence plate reader (BMG Labtech, Ortenberg, Germany).

### 2.5 Statistical analysis

All results have been presented in terms of mean and standard deviation (S.D.). Statistical analyses were performed with one-way analysis of variance (ANOVA), with the Tukey (if data were homoscedastic) or Games-Howell (if data were non-homoscedastic) post-hoc test being used for multiple comparisons with the SPSS software (SPSS Inc., Tokyo, Japan.). A *p* value of < 0.05 was considered to indicate significant differences between groups.

## 3 Results

### 3.1 Effects of PIC on *Ho-1* and *Sod1* mRNA expression

**Fig. 1:**
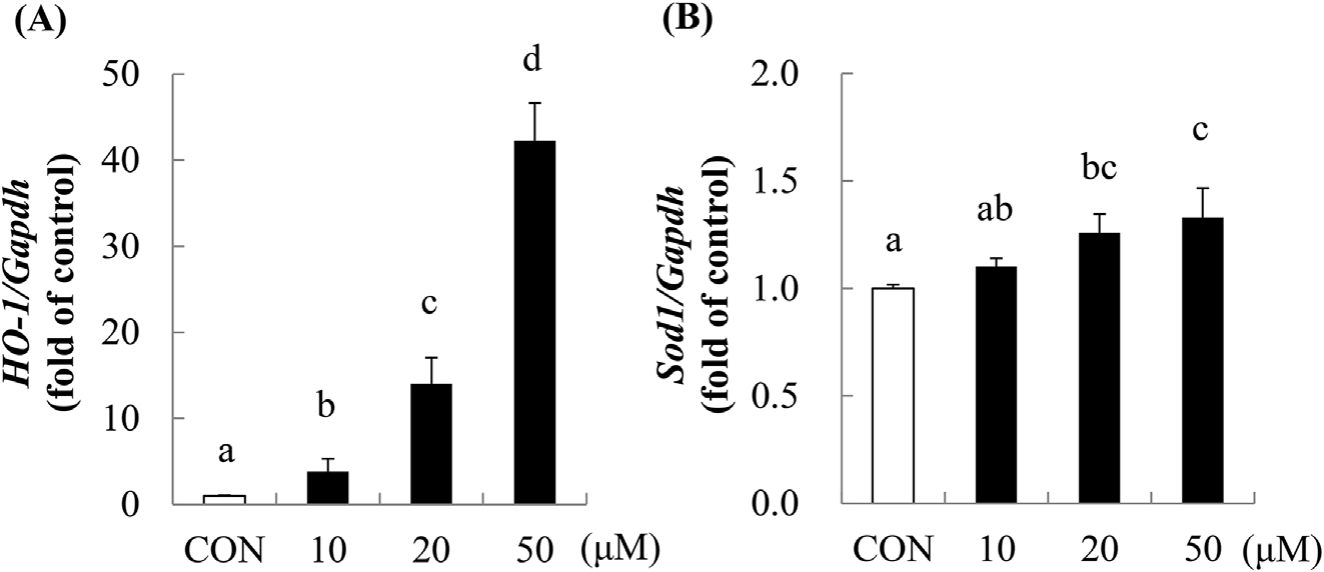
Effects of PIC on *Ho-1* and *Sod1* mRNA expression. C2C12 myotubes were incubated with the DMSO control (CON; white bar) or 10–50 μM piceatannol (black bars) for 6 h, and *Ho-1* (A) and *Sod1* (B) mRNA expression was analyzed by real-time PCR. Values have been expressed in terms of the fold change compared with the control, which was arbitrarily set to 1. Results have been provided as mean + S.D. values from at least three separate experiments. Different alphabets represent significant difference at *p* < 0.05; the analysis involved ANOVA with the Games–Howell post-hoc test (A) or Tukey post-hoc test (B).

To examine whether PIC affects the gene expression of antioxidant enzymes in skeletal muscle cells, *Ho-1* and *Sod1* mRNA expression were analyzed by real-time PCR analysis. When C2C12 myotubes were treated with 10–50 μM PIC for 6 h, *Ho-1* and *Sod1* mRNA expression was upregulated in a dose-dependent manner (Fig. 1). In particular, PIC induced marked *Ho-*1 mRNA expression: treatment with 10, 20, and 50 μM PIC upregulated *Ho-*1 mRNA expression by 3.8-, 14.0-, and 42.2-fold, respectively. Treatment with 10, 20, and 50 μM PIC upregulated *Sod1* mRNA expression by 1.1-, 1.3-, and 1.3-fold, respectively.

### 3.2 Effects of PIC analogues and major polyphenols on the mRNA expression of *Ho-1* and *Sod1*

**Fig. 2:**
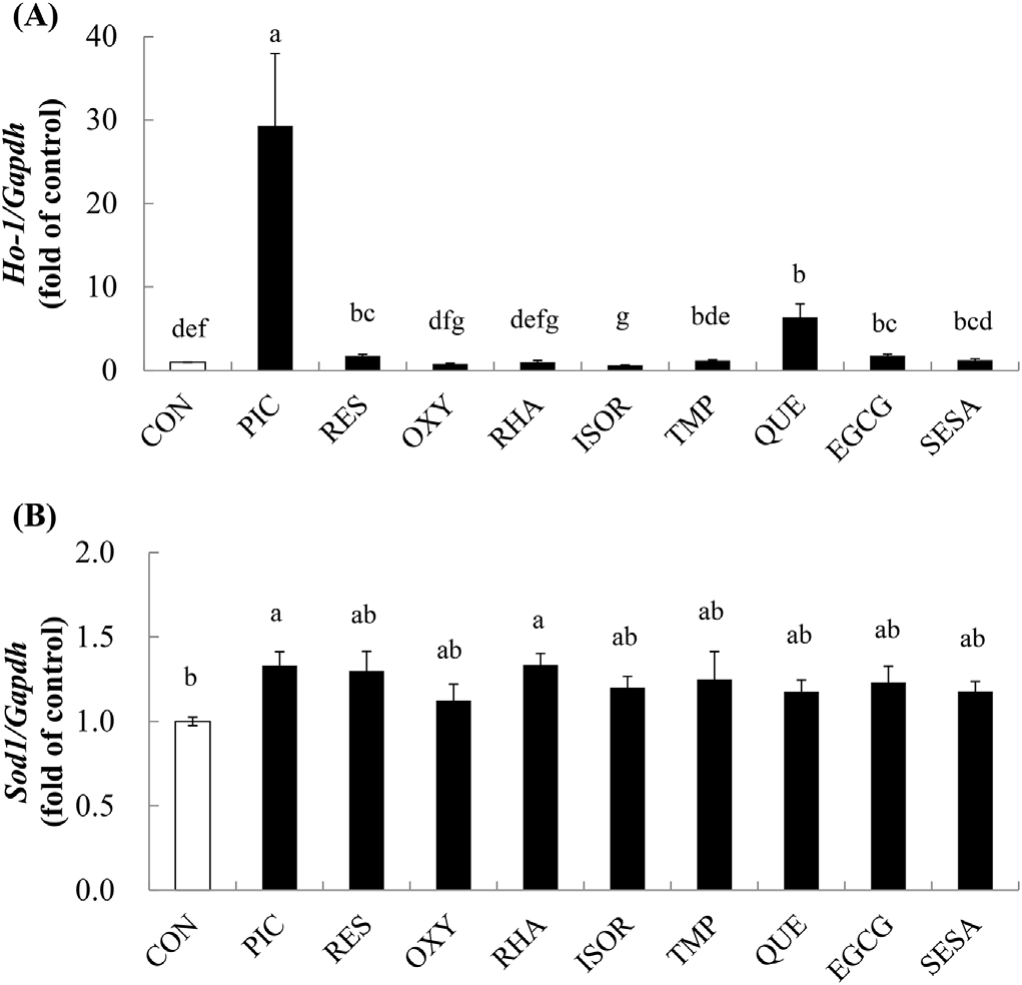
Effects of PIC and other compounds on *Ho-1* and *Sod1* mRNA expression. C2C12 myotubes were stimulated with the DMSO control (CON; white bar) or stimulants (50 μM; black bars) for 6 h, and *Ho-1* (A) and *Sod1* (B) mRNA expression was analyzed by real-time PCR. The stimulants were piceatannol (PIC), resveratrol (RES), oxyresveratrol (OXY), rhapontigenin (RHA), isorhapontigenin (ISOR), 3,3',4,5'-tetramethoxypiceatannol (TMP), quercetin (QUE), epigallocatechin gallate (EGCG), and sesamin (SESA). Values have been expressed in terms of the fold change compared with the control, which was arbitrarily set to 1. Results have been provided as mean + S.D. values from four separate experiments. Different alphabets represent significant difference at *p* < 0.05; the analysis involved ANOVA with the Games–Howell post-hoc test.

To investigate the potency of PIC against antioxidant enzyme induction, the effects of PIC on antioxidant enzyme expression were compared to those of PIC analogues and major polyphenols. Treatment with PIC, RES, QUE, and EGCG significantly upregulated *Ho-*1 mRNA expression (Fig. 2A) by 29.3-, 1.8-, 6.4-, and 1.8-fold, respectively. PIC dramatically upregulated *Ho-*1 mRNA expression, and was the most potent *Ho-1* inducer among those tested. PIC and RHA significantly upregulated *Sod1* mRNA expression by 1.3- and 1.3- fold, respectively (Fig. 2B). The *Sod1*-inducing effect of PIC was similar to that of the other compounds.

### 3.3 Effects of PIC on intracellular levels of H_2_O_2_-induced ROS

**Fig. 3:**
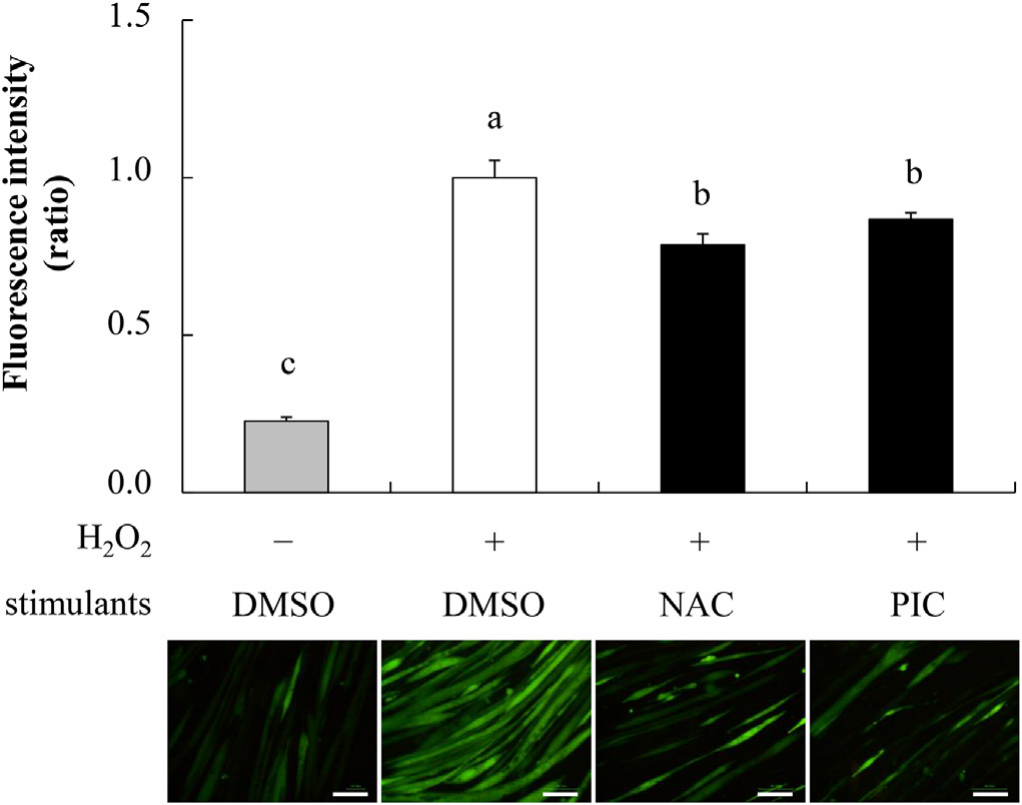
Effects of PIC on H_2_O_2_-induced intracellular ROS levels. The cells were incubated with the DMSO control, 1mM N-acetylcysteine (NAC), or 20 μM piceatannol (PIC) and loaded with 3 μM CM-H_2_DCFDA for 30 min. ROS accumulation was determined on treatment with or without 50 μM H_2_O_2_. The graph shows the fluorescence intensity produced by ROS. Values have been expressed in terms of the fold change compared with the DMSO control with H_2_O_2_, which was arbitrarily set to 1. Results have been provided as mean + S.D. values from at least four separate experiments. Different alphabets represent significant difference at *p* < 0.05; the analysis involved ANOVA with the Tukey post-hoc test. The bottom panel depicts representative fluorescence microscopy images showing the fluorescence intensity produced by ROS. The scale bar is 100 μm.

To examine whether PIC affects oxidative stress-induced ROS accumulation, the levels of ROS induced by H_2_O_2_ were measured. Addition of 50 μM H_2_O_2_ induced 4.5-fold increase in intracellular ROS levels. Pretreatment with 1 mM NAC (powerful antioxidant) and 20 μM PIC for 24 h suppressed H_2_O_2_-induced ROS production (Fig. 3), leading to a significant decrease in the ROS levels by 21% and 13%, respectively.

## 4 Discussion

In the present study, we found that (a) PIC induced antioxidant enzymes in a cultured skeletal muscle cell line, (b) PIC had superior effect on *Ho-*1 mRNA expression compared to those of PIC analogues and major polyphenols, and (c) the induction potency of PIC for *Sod1* was on the similar level with those of PIC analogues and major polyphenols. An inducer of HO-1 suppress proinflammatory cytokine and ROS production, which results in ameliorating muscle mass loss [26], therefore PIC may be an optimal polyphenol that improve muscle dysfunctions.

The induction potency for *Ho-1* differed between PIC, RES, and other PIC analogues. The mechanism underlying *Ho-1* upregulation by polyphenols has been investigated previously. Genes encoding antioxidant enzymes such as *Ho-1* exist downstream of the antioxidant response element (ARE), and their expression is mainly controlled by the Keap-Nrf2 pathway [27,28]. Some polyphenols, including PIC, activate Nrf2 signaling [20,22,29]. In particular, PIC and QUE activate Nrf2 through structural changes in Keap1. PIC and QUE have a catechol moiety; it is proposed that an electrophilic quinone is formed because of oxidation of PIC or QUE and interacts with the critical cysteine thiol of Keap1, thereby facilitating the dissociation of Nrf2 [21,30]. RES, which has no catechol moiety, did not show a strong *Ho-1* inducing effect in this study. ISOR and RHA, a structure in which one of the hydroxyl groups of the catechol group in PIC was methylated, showed no *Ho-1* inducing effects as seen when PIC was treated. No *Ho-1* induction was observed either for TMP in which all the hydroxyl groups of PIC were methylated. Therefore, these results suggest that the presence of catechol moiety strongly affects *Ho-1* expression. OXY, which has the same number of hydroxyl groups as PIC, did not show *Ho-1* induction like PIC. Therefore it is considered that the position of the hydroxyl group, not the number of hydroxyl groups, is important for *Ho-1* induction. EGCG and SESA, which is known to have high antioxidant ability, did not have strong *Ho-1* inducing effects as seen in PIC.

PIC dramatically upregulated *Ho-1* expression, though the change in *Sod1* expression was moderate. A study involving mouse embryonic fibroblasts derived from Nrf2-knockout mice revealed suppressed *Ho-1* expression and elevated *Sod1* expression [31]. Another study showed that Nrf2 knockdown in C2C12 downregulated many antioxidant enzymes such as *Ho-1* and *Catalase*, whereas *Sod1* expression did not differ from that of the wild type [32]. These results suggest that *Sod1* is not mainly dependent on the Nrf2-ARE pathway. PIC has been reported to activate the Nrf2 pathway; therefore, the difference in the contribution of the Nrf2 pathway against *Ho-1* and *Sod1* may involve the difference in induction potency of PIC for *Ho-1* and *Sod1*.

In C2C12 myoblast, inhibition of HO-1 activity dramatically abolishes the suppression of ROS accumulation [33,34]. In human endothelial cells, 12 h PIC-treatment suppresses intracellular ROS accumulation, and this effect is canceled by inhibition of HO-1 activity [23]. Therefore, upregulation of *Ho-1* expression is related to suppression of ROS accumulation in various cells. PIC induced marked *Ho-*1 mRNA expression, though the suppression of intracellular ROS by PIC was not so dramatic in this study. The highest *Ho-1* induction was observed at 6 h PIC-treatment, and ROS accumulation was examined after 24 h PIC-treatment, therefore, inhibition efficacy may be changed by the treatment time of PIC.

In summary, we showed that PIC upregulates *Ho-1* and *Sod1* gene expression and decreases H_2_O_2_-induced ROS accumulation in C2C12 myotubes. These results suggested that PIC protects skeletal muscles from oxidative stress by activating antioxidant enzymes. The findings would also be helpful in future research on prevention of oxidative stress–induced muscle dysfunction such as sarcopenia and muscle fatigue by using PIC.
